# Barriers and facilitators to primary health care for people with intellectual disabilities and/or autism: an integrative review

**DOI:** 10.3399/bjgpopen20X101030

**Published:** 2020-07-01

**Authors:** Alison Jayne Doherty, Helen Atherton, Paul Boland, Richard Hastings, Lucy Hives, Kerry Hood, Lynn James-Jenkinson, Ralph Leavey, Elizabeth Randell, Janet Reed, Laurence Taggart, Neil Wilson, Umesh Chauhan

**Affiliations:** 1 University of Central Lancashire, Preston, UK; 2 University of Warwick, Coventry, UK; 3 Cardiff University, Cardiff, UK; 4 Pathways Associates (CIC), Accrington, UK; 5 Ulster University, Coleraine, UK

**Keywords:** intellectual disability, autistic disorder, review, barriers, facilitators, primary health care, general practice

## Abstract

**Background:**

Globally, people with intellectual disabilities and/or autism experience health inequalities. Death occurs at a younger age and the prevalence of long-term morbidities is higher than in the general population. Despite this, their primary healthcare access rates are lower than the general population, their health needs are often unmet, and their views and experiences are frequently overlooked in research, policy, and practice.

**Aim:**

To investigate the barriers and facilitators reported by individuals with intellectual disabilities, autism, or both, and/or their carers, to accessing and utilising primary health care for their physical and mental health needs.

**Design & setting:**

An integrative review was undertaken, which used systematic review methodology.

**Method:**

Electronic databases MEDLINE, Embase, CINAHL (Cumulative Index to Nursing and Allied Health Literature), and Cochrane were searched for relevant studies (all languages) using a search strategy. Two researchers independently screened the results and assessed the quality of the studies.

**Results:**

Sixty-three international studies were identified. Six main themes relating to barriers and facilitators emerged from an analysis of these studies. The main themes were: training; knowledge and awareness; communication; fear and embarrassment; involvement in healthcare decision-making; and time. All the themes were underpinned by the need for greater care, dignity, respect, collaborative relationships, and reasonable adjustments. Opposing barriers and facilitators were identified within each of the main themes.

**Conclusion:**

Adolescents and adults with intellectual disabilities and/or autism experience several barriers to accessing and utilising primary health care. The findings highlight the reasonable adjustments and facilitators that can be implemented to ensure that these individuals are not excluded from primary health care.

## How this fits in

This review synthesises evidence on the barriers and facilitators to accessing and utilising primary health care perceived by people with intellectual disabilities and/or autism. The findings highlight important considerations for primary healthcare policy, practice, and further research.

## Introduction

People with intellectual disabilities and/or autism experience health inequalities.^1,2^ Death occurs at a younger age and the prevalence of long-term morbidities is higher than in the general population.^2^ UK guidance outlines the necessity of tailoring healthcare services to meet their needs.^3^ Despite this, their access to healthcare services is lower than the general population,^4,5^ their health needs are often unrecognised or unmet,^6^ and their views and experiences are frequently overlooked.^7,8^

While previous reviews have investigated healthcare-access issues for people with intellectual disability,^9,10^ a recent mapping of the health and wellbeing needs of adults with both intellectual disability and autism identified an absence of research to determine their needs.^11^ This lack of understanding represents a significant knowledge gap in efforts to improve their health and wellbeing. There may be overlap between these groups, but their needs may be unique and nuanced.^11^

The aim of this study was to identify and synthesise evidence concerning the barriers and facilitators experienced by adolescents and adults with intellectual disabilities (only), autism (only), or both, and/or their carers, to accessing and/or utilising primary health care for their physical and mental health needs.

## Method

**Figure 1.**
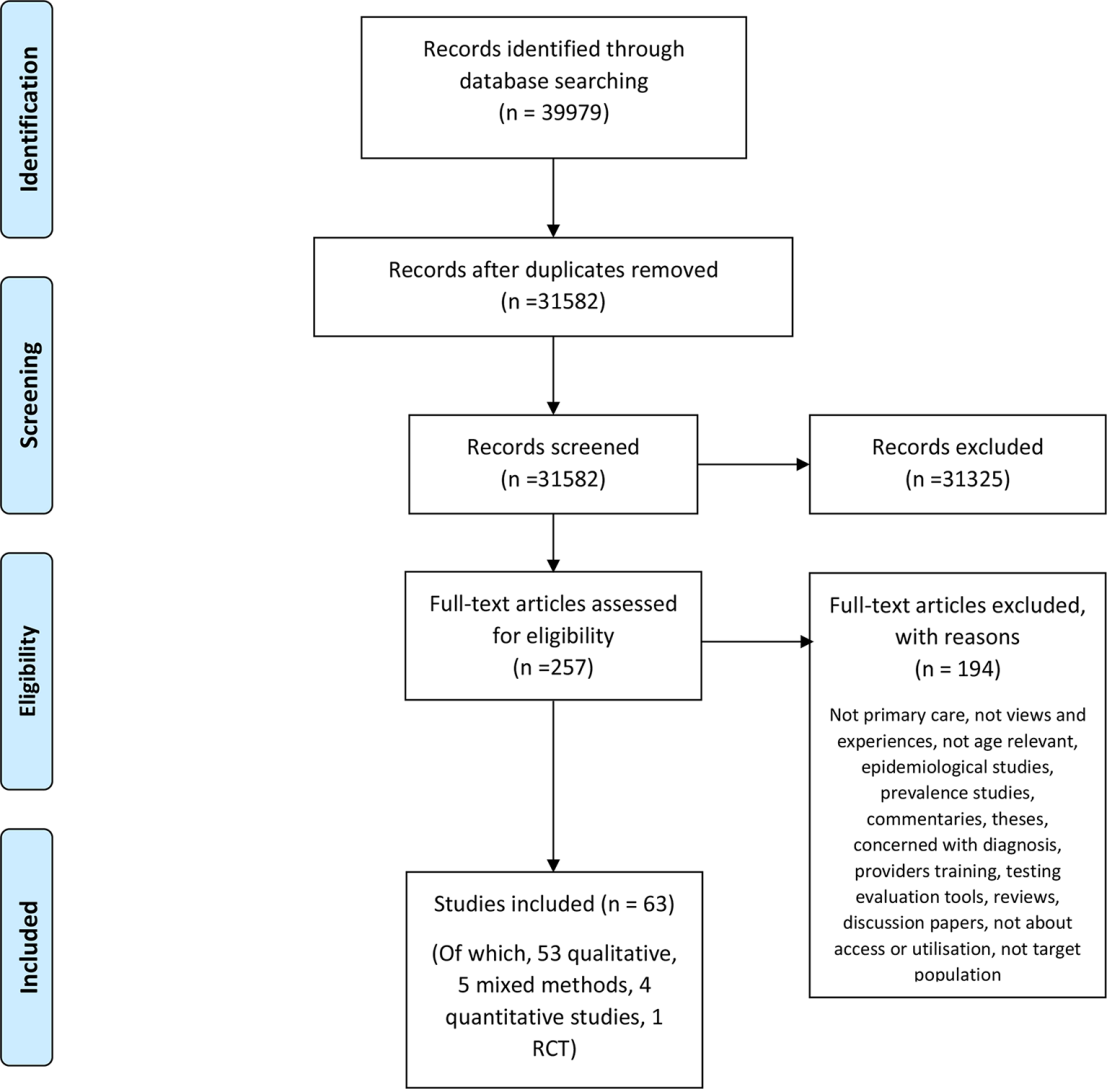
PRISMA diagram illustrating the literature search strategy. RCT = randomised controlled trial.

The integrative review (utilising systematic review methodology) was conducted according to a pre-specified protocol and written in accordance with the Preferred Reporting Items for Systematic Reviews and Meta-Analyses (PRISMA) 2009 checklist and reporting standards (Figure 1).^12^ The search strategy is contained within the registered protocol. Electronic databases were searched using key terms and MeSH headings combined. The databases used were: Ovid Medline (up to 22 August 2018); Embase (22 August 2018); CINAHL Complete (22 August 2018); and Cochrane (22 August 2018).

Studies were eligible if they were: studies of any design; and included people aged ≥14 years (eligible for annual health checks in UK) who were formally identified, or self-identified, as having an intellectual disability and/or autism, and their family members, carers, support workers, and/or healthcare professionals. Studies were also eligible that explored access to health care for any physical and/or mental health conditions involving the target population. The studies could be from any primary healthcare setting; for example, GP practices and other providers, pharmacies, dental surgeries, ophthalmic services, and screening and immunisation services. Studies included primary healthcare services in the UK and in other countries with similarly structured, funded, and resourced services. Eligible studies were published in all languages between 2001 and 2018. These publication dates were not stated in the study protocol. Studies conducted before 2001 were excluded owing to legislative changes introduced for the target population, including the Department of Health and Social Care’s *Valuing People* *—* *A New* *Strategy* *for Learning Disability for the 21st Century*, published in 2001.^13^ Systematic reviews, book reviews, editorials, commentaries, epidemiological studies, and prevalence studies were excluded.

‘Access to health care’ in this review is concerned with helping people to command appropriate healthcare resources to preserve or improve their health, and ‘equity of access’ is considered in terms of availability, utilisation, or service outcomes.^14,15^

Titles and abstracts were first screened for relevance independently by two researchers. Three researchers independently read and assessed the full texts of relevant citations, using the pre-specified eligibility criteria. Any citation queries were discussed by the three researchers and a consensus decision reached to resolve any queries.

Study quality was assessed independently by two researchers using the Mixed Methods Analysis Tool (MMAT).^16,17^ The MMAT is designed for use in the appraisal stage of systematic reviews of different types of studies. The critical appraisal of studies considered issues such as the appropriateness of the study’s design to the study’s research objective. This was used to provide context for the findings of the study. The two researchers independently assessed the number of criteria met by each study in each of the MMAT’s domains of assessment^17^ and provided each study with a score. Studies were rated as high quality (*****) if all of the MMAT criteria were met, good quality (****) if 75% of the criteria were met, satisfactory (***) if 50% of the criteria were met, poor (**) if the study met 25% of the criteria, and very poor (*) if the study met <25% of the MMAT scoring criteria. The researchers’ independent appraisal findings were compared and agreed. Any queries over studies’ appraisals were discussed with two other independent researchers to reach a consensus decision.

Data were extracted from the included studies using a data extraction tool, which was specifically designed and piloted by the research team. The data from the included studies were analysed using thematic analysis.^18^ Two researchers independently developed the themes. These themes were compared, key themes agreed, and narratively synthesised by the two researchers. A third reviewer was involved where necessary. NVivo (version 12) supported the data analysis.

## Results

Sixty-three studies met the eligibility criteria for inclusion in the review. The review identified 53 qualitative studies, five mixed-methods studies, four quantitative studies, and one randomised controlled trial. Twenty-four studies were conducted between the years 2003 and 2010, and 39 studies were conducted between 2011 and 2018. Most studies were conducted in the UK (*n* = 33), the US (*n* = 13), and Australia (*n* = 8). Forty-nine studies explored the views and experiences of participants with intellectual disabilities, 13 studies explored the views and experiences of participants with autism, and one study explored the views and experiences of participants with both intellectual disabilities and autism.

### Quality of studies

Of the 63 eligible studies, 46 (73%) were rated as high quality, two were rated as good quality, seven were rated as being either satisfactory, poor, or very poor, and eight were not rated owing to their lack of reported information. However, none of the studies were excluded from the review as any appraisal process is potentially only evaluating the reporting of the study rather than its actual conduct and content, which may usefully inform the findings and discussion.^19^

### Themes

Six common themes were identified by the thematic analysis. Participants perceived of barriers and facilitators within each of these themes as being integrated in an opposing fashion, as narratively discussed below.

#### 1. Training

Healthcare providers (both primary and acute health care) may lack specialist training in this field.^20–22^ Barriers to their training include time constraints, knowledge gaps, and uncertainties over specialist help.^23^ Despite this, healthcare providers recognise the importance of such specialist training for people with intellectual disabilities and for others with communication challenges.^23^ Training, knowledge, and awareness-raising for healthcare providers, family members, carers, and support services is essential;^24^ for example, training for healthcare providers in communicating with people who have intellectual disabilities and/or autism.^25^ People with intellectual disabilities should be included in the training of healthcare providers as ‘experts by experience’.^25–27^ However, while such training may be essential, it may not be routinely undertaken owing to resource constraints.^22^

#### 2. Knowledge and awareness

Some healthcare providers may lack understanding, knowledge, and awareness about to how to support people with autism and intellectual disabilities,^28–30^ including how to make appropriate reasonable adjustments.^29^ This lack of knowledge and understanding may lead to poor attitudes, such as an abrupt way of speaking or coldness towards people with autism or intellectual disabilities. Poor attitudes held by both healthcare professionals and non-health professionals, across both primary and acute healthcare provider settings, is a recurring theme in the review’s identified studies.^22,31–33^ A warm, friendly, and caring attitude from healthcare providers enables service users to access healthcare facilities and discuss sensitive health concerns.^22,34–37^

#### 3. Communication

Communication is a significant barrier for people with autism and/or intellectual disabilities.^29,38–42^ It causes problems in primary care as inadequate communication can result in the wrong diagnosis and inappropriate medication, and it can prevent a person’s access to receiving adequate health care.^23,43,44^ Studies found a lack of awareness by healthcare providers about the range of communication issues faced by people with intellectual disabilities and/or autism when accessing and attending primary and acute healthcare settings.^23,45–47^ Healthcare practitioners may rely on communicating with a carer, family member, or support worker rather than with the service user directly.^23,25,36,45,47^ Carers do not always allow the service user to speak for themselves or carers might try to protect them from perceived harmful communication,^28^ thereby preventing service users from exerting control over their own healthcare needs.^32^ Not being listened to created anxiety for some service users with intellectual disabilities and/or autism.^48^

Good communication between the healthcare provider and the patient with autism and/or an intellectual disability is vital when accessing and utilising health care.^48,49^ If these patients find health care stressful because of poor communication then they may lower their expectations, lower their attendance, and feel disaffected, and this may lead to ineffective health care.^23^

Some healthcare information may be incomprehensible and/or difficult to obtain.^25,34,50,51^ Accessible healthcare information is perceived to be a high priority by people with intellectual disabilities and/or autism.^48–50^ The use of easy-read information, sign language, non-face-to-face communication, such as via the telephone, not overloading the service user with verbal information, and use of virtual reality have been suggested as preferred methods of communication for this population.^26,52^

The ability to see the same healthcare professional is important for people with intellectual disabilities and/or autism.^34^ This notion is also shared by healthcare professionals themselves, with suggestions that this would provide the opportunity to gain a better understanding of the medical history and communication style of the service user.^36^ Being treated with dignity and respect and being valued by healthcare providers are key facilitators,^47,53^ and are important in forming good relationships.^54^ Collaborations between health and other social care providers are also essential.^55,56^

#### 4. Fear and embarrassment

Fear and embarrassment is a barrier to accessing health care for individuals with intellectual disabilities and/or autism.^57–59^ These include fears of being judged over lifestyle choices, of blood tests and vaccinations,^38^ of medical instruments,^31^ and fears associated with a lack of understanding about screening procedures.^59^ Physical examinations can also be a source of embarrassment and/or discomfort for individuals with intellectual disabilities.^38,60–63^ Some people with intellectual disabilities and/or autism find the clinical environment daunting,^63,64^ owing to unpleasant or alarming noises, odours, and bright lights.^31,65^ The waiting room may induce anxiety, especially if the individual is unsure of why they are there.^32^ Facilitators, in terms of reasonable adjustments in the clinical environment, may include easy-read information, coloured pictures, models, photos, videos, symbols, and demonstration dolls.^13,36,48,65^

#### 5. Lack of involvement in healthcare decision-making

People with intellectual disabilities and/or autism can make choices about their everyday lives, yet decisions about their health care may be made by their families, carers, and healthcare providers instead.^25^ Their involvement in the decision-making process is an essential part of their health care.^13,34^ They feel empowered when they are involved in the decision-making process and gain a better understanding of their treatment and diagnosis.^28,66^

People with intellectual disabilities and/or autism value healthcare professionals, their support network, and other professionals who work closely with them and who have specialist knowledge and experience of working with people who have intellectual disabilities.^26,67–72^ A joined-up approach, in which the sharing of inter-agency information is key, may help alleviate service users’ healthcare fears.^26,36^ Tailored services, which are person-centred, flexible, and family-centred, are highly regarded.^26,72–77^

#### 6. Time

Prolonged times waiting to be seen and limited time spent with a healthcare professional during an appointment act as barriers. Long waiting times are a major cause of anxiety and stress.^25,32,45,78,79^ Additional time is often required for effective communication with people who have intellectual disabilities and/or autism.^31,36,47,48^ However, despite recognising the importance of spending time getting to know the service user, some healthcare providers can struggle to find extra time to achieve this familiarisation.^22^

## Discussion

### Summary

A lack of specialist training in both primary and acute health care is an important barrier, which may mean that healthcare providers lack knowledge and awareness of the healthcare needs of people with intellectual disabilities and/or autism. This may be associated with poor communication between such healthcare providers and service users, and a lack of involvement in healthcare decision-making processes for these service users. Effective communication delivered by specialist trained, friendly, and caring healthcare providers who treat service users with dignity is essential. Primary healthcare providers need to provide more accessible health-related information for service users (both in terms of availability and format), shorter waiting times and longer consultation times, less daunting clinical environments, improved consistency of care, and greater multidisciplinary collaborative working. This may help to improve the health and wellbeing of people with intellectual disabilities and/or autism. Improving these issues could help alleviate some of the fears reported by service users, which represent another reported barrier. However, the challenges involved in responding to these identified issues are acknowledged given that primary healthcare services in the UK are currently under intense resourcing pressures.^80^

### Strengths and limitations

A strength of this review is that it provides a timely summary of the recent literature from 2001–2018. The review, importantly, included the views and experiences of people with intellectual disabilities and/or autism, and their families or carers, as well as healthcare professionals. It synthesised different types of studies using a rigorous methodology. However, a search of other relevant databases, such as PsycINFO, grey literature (including guidance and policy documents), and checking the references and citations of included studies, may have yielded additional results. Searches of grey literature and the checking of reference lists and citations for included studies were not undertaken, as originally stated in the study protocol, owing to staffing resource constraints.

Most of the identified studies were conducted in high-income countries (HICs) and may not reflect the views and experiences of people with intellectual disabilities and/or autism from low- and middle-income countries. Findings are not generalisable to all HICs either, as the review included studies from HICs such as the US, with different funding and organisational arrangements. The review was limited to studies involving individuals with intellectual disabilities and/or autism who were aged ≥14 years. There may be transferable evidence from studies involving children and younger people from this population and other cognitive disability populations (for example, patients with dementia, cerebral palsy, stroke, or acquired brain injury). The use of convenience samples^23,56^ and self-selection^20^ may also bias the results. Some studies were limited to urban areas as opposed to rural areas, which may pose different healthcare barriers.^81,82^

### Comparison with existing literature

To the authors’ knowledge, this is the first integrative review of barriers and facilitators to accessing and utilising primary health care experienced by adolescents and adults with intellectual disabilities and/or autism. The review’s findings are consistent with available UK guidance for GPs and other primary healthcare professionals, which outline the need to tailor primary care services for people with intellectual disabilities.^3^

### Implications for research and practice

The review’s findings highlight the reasonable adjustments and other modifications that could be implemented to ensure that people with intellectual disabilities and/or autism are not excluded from primary healthcare research and practice. Despite the constraints facing primary healthcare services in countries such as the UK, their contributions are crucial in addressing the health inequalities experienced by this population.

## Trial registration number

PROSPERO: CRD42018103103.
